# Plasticity of empty major histocompatibility complex class I molecules determines peptide-selector function

**DOI:** 10.1016/j.molimm.2015.03.010

**Published:** 2015-12

**Authors:** Andy van Hateren, Alistair Bailey, Jörn M. Werner, Tim Elliott

**Affiliations:** a Institute for Life Sciences, Building 85, M55, University of Southampton, SO17 1BJ, UK; b Cancer Sciences Unit, Faculty of Medicine, University of Southampton, SO16 6YD, UK; c Centre for Biological Sciences, Faculty of Natural & Environmental Sciences, Building 85, M55, University of Southampton, SO17 1BJ, UK

**Keywords:** MHC class I, Tapasin, Protein plasticity, Peptide selection, Peptide editing

## Abstract

Major histocompatibility complex class I (MHC I) proteins provide protection from intracellular pathogens and cancer via each of a cell's MHC I molecules binding and presenting a peptide to cytotoxic T lymphocytes. MHC I genes are highly polymorphic and can have significant diversity, with polymorphisms predominantly localised in the peptide-binding groove where they can change peptide-binding specificity. However, polymorphic residues may also determine other functional properties, such as how dependent MHC I alleles are on the peptide-loading complex for optimal acquisition of peptide cargo. We describe how differences in the peptide-binding properties of two MHC I alleles correlates with altered conformational flexibility in the peptide-empty state. We hypothesise that plasticity is an intrinsic property encoded by the protein sequence, and that co-ordinated movements of the membrane-proximal and membrane-distal domains collectively determines how dependent MHC I are on the peptide-loading complex for efficient assembly with high affinity peptides.

## Highlights

• MHC class I alleles vary in their intrinsic ability to select optimal peptides.
• Ability of MHC to self-assemble is inversely correlated with dependence on tapasin.
• Variation in peptide selector function correlates with the plasticity of empty MHC.
• Increased plasticity of empty MHC I allows more efficient peptide selector function.
• Co-ordinated domain–domain movements contribute to determine plasticity.

## 1 MHC I alleles differ in dependence upon tapasin and the peptide-loading complex

Peptides presented at the cell surface by MHC I molecules help to protect the host from intracellular pathogens and tumours. These peptides are generated in the cytoplasm by the proteasome and other proteases, and are translocated by the transporter associated with antigen processing (TAP) into the endoplasmic reticulum (ER) where MHC I peptide-loading occurs. A TAP-associated peptide-loading complex (PLC), of which the cofactor tapasin is a key constituent, ensures MHC I molecules efficiently load with peptides. Once assembled, peptide-MHC I complexes pass through the secretory pathway to populate the cell surface. Here the peptides are presented to cytotoxic T lymphocytes which discriminate between healthy and abnormal cells. Which peptides are selected for presentation by MHC I is therefore of fundamental importance for modulating the immune response.

MHC I peptide selection is thought to occur in two stages (Lewis and Elliott, 1998), with empty MHC I molecules initially binding whatever peptides are likely to be the most abundant in the ER. This cargo is unlikely to bind with high affinity as few peptides transported by TAP will match the peptide-binding specificities of the MHC I alleles present. Although this initial peptide cargo fails to elicit an effective immune response because it does not sufficiently stabilise MHC I (Lewis and Elliott, 1998), a low affinity cargo will prevent the rapid and irreversible inactivation that MHC I undergoes in the absence of peptide. MHC I molecules subsequently exchange this sub-optimal cargo for high affinity peptides in a process catalysed by tapasin and the PLC. Tapasin facilitates MHC peptide-loading in multiple ways: tethering unloaded or poorly loaded MHC I to the TAP peptide portal (Sadasivan et al., 1996); stabilising TAP and consequently increasing the rate of peptide translocation (Grandea et al., 2000, Lehner et al., 1998); synergistically strengthening the interactions between components of the PLC (van Hateren et al., 2010); perhaps most importantly tapasin improves the rate and the extent of peptide loading and the discrimination that occurs between peptides (Williams et al., 2002, Wearsch and Cresswell, 2007). This peptide selection function results in MHC I becoming loaded with those peptides that bind with high affinity, prolonging cell surface expression (Howarth et al., 2004).

A survey of the assembly properties of common North American HLA B alleles revealed a hierarchy in which the intrinsic ability to select and assemble with optimal peptides is inversely correlated against the extent that tapasin enhances MHC I peptide loading (Rizvi et al., 2014). This observation of an inverse correlation is consistent with earlier studies showing human MHC I alleles require tapasin and the PLC to different extents for efficient peptide-loading (Greenwood et al., 1994, Peh et al., 1998). Like their human counterparts, the MHC I alleles of rodents differ in tapasin dependence: with murine H2-K^b^ molecules being more reliant on tapasin than H2-D^b^ molecules (Garbi et al., 2000). Additionally rat RT1-A^u^ molecules can gain an optimal peptide repertoire independently of the PLC when RT1-A^a^ molecules are over-expressed, illustrating that RT1-A^u^ possess significant intrinsic ability to self-select and assemble with high affinity peptides (Ford et al., 2004).

Various arguments have been proposed to explain why diversity in assembly pathways exists. As components of the PLC are frequently inhibited by viral subversion proteins or their expression down-regulated following oncogenic transformation, there is an obvious advantage if MHC I alleles are able to load peptides efficiently without the assistance of tapasin and the PLC. An alternative and not mutually exclusive argument is that tapasin-dependent MHC I loading allows the evolutionary appearance of new peptide-binding domains that accommodate new pathogenic peptides, because tapasin may ameliorate the potentially disruptive effect that changes in the peptide-binding domain might entail (van Hateren et al., 2013).

Understanding why MHC I alleles differ in this way has been intensively researched, particularly the HLA B*44:02 and B*44:05 alleles (B4402 and B4405 hereafter) which differ only at position 116 (B4402: Asp116, B4405: Tyr116) and may define extreme ends of this phenotype. While B4402 relies upon tapasin and the PLC to assemble with high affinity peptides, B4405 efficiently self-selects high affinity peptides (Zernich et al., 2004). There is no obvious structural correlate of this difference in function, but short molecular dynamics (MD) simulations have suggested that B4402 and B4405 differ in their conformational flexibility in the peptide-empty state (Sieker et al., 2007, Garstka et al., 2011, Ostermeir et al., 2015), despite near identical static structures of the peptide bound state solved by X-ray diffraction (Zernich et al., 2004).

**Figure 1a:**
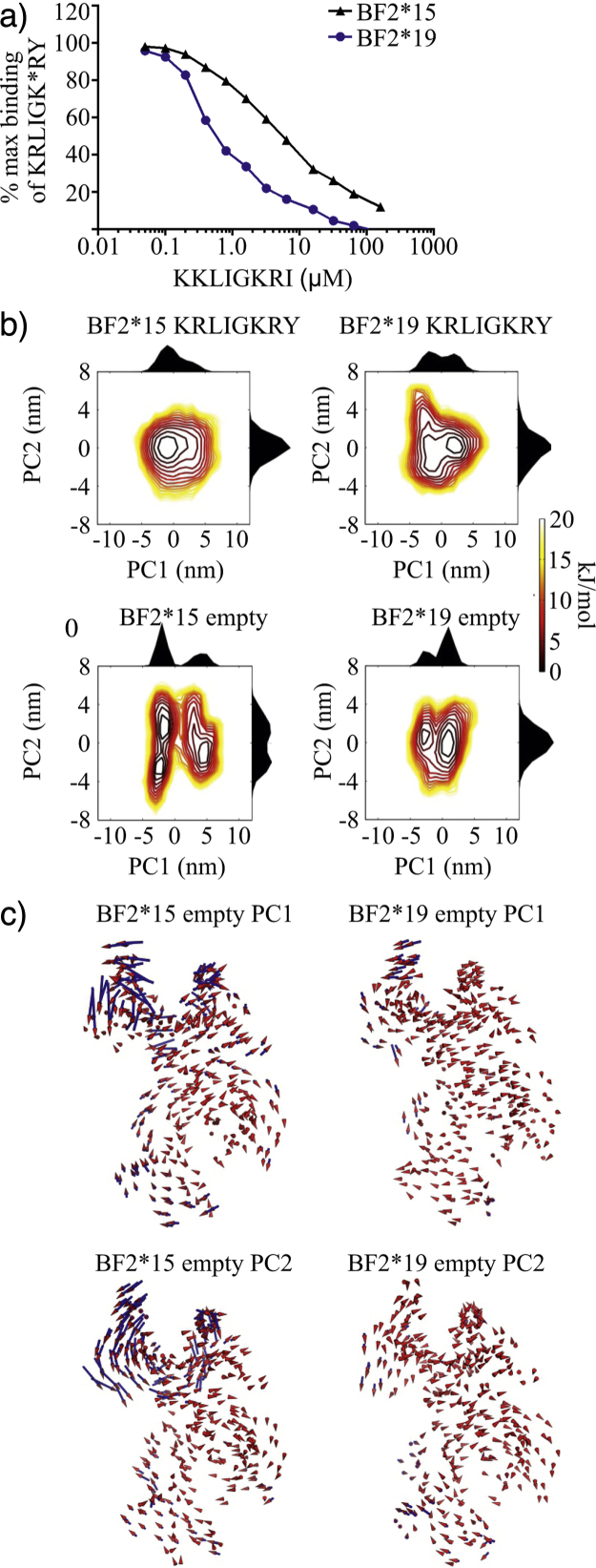
(a) BF2*15 and BF2*19 differ in their intrinsic ability to select high affinity peptides. A final concentration of 0.5 μM BF2*15 or BF2*19 molecules refolded with UV conditional ligand (produced as in van Hateren et al., 2013) were exposed to ∼360 nm light for 20 min at 4 °C in the presence of 10 μM chicken β_2_-microglobulin. The UV-exposed proteins were then added to 0.1 μM high affinity peptide KRLIGK*RY (K* represents 5′ Tamra labelled Lysine) mixed with various concentrations of the low affinity competing peptide KKLIGKRI (0–160 μM) in a total volume of 60 μl. Fluorescence polarisations measurements were taken after being left at room temperature overnight as described in van Hateren et al. (2013) and plotted as a percentage of the maximum polarisation value (observed in the absence of KKLIGKRI). (b) BF2*15 and BF2*19 differ in their abilities to explore the conformational landscapes in the peptide-empty state. Gibbs free energy landscapes were generated from the principal coordinates of Principal Component PC1 and Principal Component PC2 and transformed by treatment as a Boltzmann ensemble as in Bailey et al. Individual probability densities for PC1 and PC2 are plotted on the outside adjacent axes. (c) The global dynamics of MHC I identified by Principal Component Analysis. 420 nanosecond MD simulations of BF2*15 and BF2*19 were performed using a common peptide free backbone structure as in Bailey et al. Porcupine plots indicate the magnitude and direction of motion for each backbone atom along Principal Component PC1 (top panels) and Principal Component PC2 (bottom panels) in the peptide free state. The arrows indicate the direction of the motion of each atom along this mode. The length of the arrow tail indicates the magnitude of the motion.

Our comparison of the peptide-binding properties of chicken MHC I alleles (van Hateren et al., 2013), separated from a common ancestor of humans by ∼350 million years, has shown that these differences in MHC I peptide-binding properties and assembly pathways are an evolutionarily ancient property. We found that the chicken MHC I molecules BF2*15:01 and BF2*19:01 (BF2*15 and BF2*19 hereafter), which differ by only eight amino acids and bind a similar peptide repertoire, differ in their peptide-binding properties (van Hateren et al., 2013), and thus show similarities to human alleles (Rizvi et al., 2014). We found BF2*15 was similar to B4405 and certain other alleles that have greater intrinsic ability to select and assemble with high affinity peptides than alleles such as BF2*19, B4402 and other “poor self-assembling” molecules. Consistent with the correlation described in humans (Rizvi et al., 2014), BF2*19 receives greater enhancement from tapasin than BF2*15. Lastly we have since shown that BF2*15 discriminates between high and low affinity peptides to a greater extent than BF2*19 (Fig. 1a), reminiscent of the greater intrinsic ability of good self-assembling molecules to select high affinity peptide (Williams et al., 2002).

## 2 Variable protein plasticity may explain the different peptide-selector function

In the absence of a structural correlate which may account for the variable tapasin dependence of MHC I alleles, we compared the dynamic behaviour of the ER luminal domains of BF2*15 and BF2*19 during ∼0.5 μs MD simulations, seeking to provide a mechanistic basis which may account for the differences observed in the peptide-binding properties.

Focusing initially on the peptide-bound proteins we compared the flexibility of each individual residue within the two BF2 alleles by measuring the variation of the backbone conformational angle *φ*. This dihedral angle provides a measure of the local flexibility of each residue in the protein in terms of rotation around the N-Cα bond. We found in the peptide-loaded state both proteins had a similar number of sites of local flexibility, with unstructured loop regions showing greatest variation in flexibility in both proteins.

We next examined the global motions of the peptide-loaded BF2 alleles by performing Principal Component Analysis (PCA) of the covariance of displacements of the backbone atoms during MD simulations. PCA transforms the variance of individual atomic fluctuations generated by the simulation into collective motions, ranked according to their contribution to the total backbone fluctuations. Therefore the first principal component (PC1) describes the collective motion that accounts for the greatest proportion of the total variance, and the second principal component (PC2) describes the next highest proportion. We found that the two largest principal components accounted for around a third of the total variance for both proteins. As in the *φ* angle analysis we found similarities between the peptide-loaded BF2 alleles, with the proteins exhibiting similar magnitudes of atomic fluctuations when the motions of the top two principal components were considered. Also, the extent and directions of the motions that the top two principal components describe were similar for both proteins. We observed that the α helices flanking the peptide-binding groove rolled slightly and the entire peptide-binding domain twisted or flexed relative to the membrane-proximal α_3_ domain. The similarity in the conformational flexibility of these BF2 alleles in the peptide-bound state was further confirmed by creating free energy landscapes from the top two principal components (Fig. 1b top panels). We found both peptide-loaded proteins predominantly occupied a single conformation and rarely explored other conformations. This is consistent with the numerous similar, but not identical, structures of MHC I molecules that are loaded with disparate peptides, or are from different species. Thus using a number of analyses we found little difference between the BF2 alleles during peptide-loaded MD simulations, also consistent with the peptide being bound by these alleles with equal affinity (van Hateren et al., 2013).

We next removed the peptide from these BF2 alleles and repeated the MD simulations. Although the simulations were not based on observed protein structures, the protein conformation was relatively stable during the simulations suggesting no aberrant dynamic events occurred. In contrast to the similarities described above for the peptide-loaded alleles, we found marked differences between the BF2 alleles when the peptide-empty MD simulations were analysed. The *φ* angle analysis revealed that there are more sites of local flexibility in the peptide-empty simulations compared to the peptide-loaded simulations. This is consistent with numerous pieces of indirect evidence including a study in which peptide-empty MHC molecules were suggested to possess properties similar to molten globules (Bouvier and Wiley, 1998). Intriguingly there was a twofold greater increase in the number of sites of local flexibility when empty BF2*15 was compared to peptide-loaded BF2*15 than there was for BF2*19. We also found the empty BF2 alleles differ in their abilities to explore the conformational landscapes described by the top two principal components, with BF2*15 occupying three distinct energy minima, and thus exploring more of the energy landscape than BF2*19 which predominantly occupied a single energy minimum (Fig. 1b bottom panels). Additionally we found differences between the empty BF2 alleles in the magnitude of the global motions described by the top two principal components, which was much greater for empty BF2*15 than for empty BF2*19 (Fig. 1c). Interestingly the F pocket of BF2*15 fluctuated to a much greater extent than was apparent for BF2*19, which was less mobile than in the peptide-bound state (Fig. 1c).

Collectively this suggests these BF2 alleles differ in the peptide-empty state, with empty BF2*15 having greater plasticity than empty BF2*19. BF2*15 has more sites of local flexibility, more frequently adopts different conformations, which involve larger movements, and has greater conformational flexibility concentrated around the F pocket. When our simulations are considered alongside our experimentally observed findings (van Hateren et al., 2013), it is apparent that variation in the plasticity of empty MHC I molecules correlates with their intrinsic peptide selection abilities. We hypothesise that increased plasticity of empty MHC I allows for more efficient sampling of the pool of peptides in the ER (the peptidome) in the absence of tapasin. In contrast to peptide free MHC I molecules, peptide loaded MHC I molecules displayed similar degrees of plasticity, consistent with the stabilisation of MHC I that occurs upon peptide binding, as observed experimentally (e.g. Williams et al., 2002, Saini et al., 2013).

## 3 Co-ordinated domain–domain movements may be central to MHC I plasticity

One feature we noted during the MD simulations is that flexibility is not confined to the peptide-binding domain, and that both the membrane-proximal α3 and the peptide-binding domains move during MD simulations. The domains do not move independently of each other, but instead in a co-ordinated way. This may indicate these domains are dynamically coupled, and that peptide-binding properties are collectively defined by the entire MHC I protein and not by just the peptide-binding domain. This possibility is supported by our finding that exchanging the single polymorphic residue that is located in the α3 domain between BF2*15 and BF2*19, (BF2*15: Gln220, BF2*19: Arg220) altered the peptide-binding properties of both MHC I alleles (van Hateren et al., 2013). This indicates that a functional relationship exists between position 220 and the peptide-binding domain, a possibility which is further supported by the identification of position 220 as part of an allosteric protein network through which local protein movements may be propagated throughout the protein.

We observed both BF2 alleles have increased local flexibility in the α3 domain around position 220 in the peptide empty state. This is interesting because BF2 residues 218–225 are likely to form an interaction site for tapasin (van Hateren et al., 2010, Dong et al., 2009), therefore polymorphisms located at this membrane-proximal interaction site may influence the ability of tapasin to enhance peptide selection. At least two lines of evidence support this possibility; exchange of residue 220 between BF2*15 and BF2*19 influenced the magnitude of tapasin function when tapasin and BF2 were artificially tethered together (van Hateren et al., 2013); human tapasin was unable to function when tethered to an HLA-B*08:01 mutant which has Glu222 mutated to Lys222 (Chen and Bouvier, 2007). These results are consistent with a model in which the membrane-proximal interaction may allow the peptide-binding domain to more readily adopt those conformations that allow peptide-selection to occur. Intriguingly comparison of chicken tapasin alleles reveals polymorphisms located near this membrane-proximal interaction site, supporting the possibility that chicken tapasin and BF2 have co-evolved with polymorphisms at these positions influencing MHC I peptide selection in an allele specific way via an allosteric relay (van Hateren et al., 2013).

## 4 Concluding remarks

Our finding that the ability of two chicken MHC I alleles to select and assemble with optimal peptides is inversely correlated with the magnitude of tapasin function that they experience (van Hateren et al., 2013) is consistent with observations from human HLA B alleles (Rizvi et al., 2014). This suggests that variation in peptide-selection and assembly properties is an intrinsic and evolutionarily conserved MHC I property. We hypothesise that protein plasticity underpins the intrinsic ability of empty MHC I molecules to select and assemble with optimal peptide (van Hateren et al., 2013). We believe that a network of interacting residues stretching from the tapasin-binding site in the membrane-proximal α3 domain to the peptide-binding domain contributes to determine intrinsic peptide-binding properties via co-ordinated domain–domain movements. Tapasin, which interacts with MHC I via both the membrane-proximal and membrane-distal domains, could exploit this allosteric network to enhance MHC I peptide-selection and assembly. Furthermore, we expect the plasticity of MHC I molecules to be important for other aspects of their function, perhaps for recognition by T cell receptors, CD8, or NK cell receptors or may contribute towards regulation of endocytosis.
